# The Importance of Choosing the Indicated Remedy in the Minimum Dose

**Published:** 1890-09

**Authors:** F. C. Hood

**Affiliations:** Marysville, Cal.


### THE IMPORTANCE OF CHOOSING THE INDICATED REMEDY IN THE MINIMUM DOSE.

F. C. Hood, M. D., Marysville, Cal.

How sad the environment of many a one in his preceptor's office, and in college, acting in the capacity of a student, seeking a medical education, the best he thinks the school of Homœopathy can afford. Later on in life, he proves by a practical test of the law of Homœopathy that he was reared in a crude atmosphere, educated in a medical college only a few steps in advance of the crude. He begins to practice Homœopathy with crude ideas of how to find the indicated remedy. He is confident he can cure every case he gets, and he solicits patronage in his new field with that inspiring thought, as well as impressing upon the minds of his adherents that he can cure every case, and hence financial success is certain. Alas! in a short time he has a patient who has been treated by every allopathic physician in town without improvement. He finds the indicated remedy and gives it low and fails. Thinking perhaps it was not the right remedy, he gives another and another, tries, tries and tries again, till his patient gives up in despair and he is lost. He is discouraged, his crude ideas have given him bitter disappointment. The high esteem and respect he had for his professors as being men of profound ability is now in doubt. He is left alone to experiment and to put the law of Homœopathy to a practical test. Five years ago I came to Marysville, a malarial district, and to my astonishment I found an army of little children of the allopathic persuasion, pale, sallow, and ghostly in appearance, following the advice of their physicians, namely taking Quinine for breakfast, Fowler's solution for dinner, and Calomel for supper. I visited many families; was informed that everybody kept Quinine on the mantel-piece and everybody took it. I also inquired of several homœopathic families and they too said everybody takes Quinine because it is the only specific. Homœopathic physicians have to give it to cure their patients as well as the old school.

In a short time I was called twelve miles into the country to visit Mrs. E., who was suffering with intermittent fever. Chill came on at eleven A. M., preceded by severe bone pains, ringing in the ears; wanted to drink often and large quantities; fever high; mild delirium; sweat sticky and very profuse; sallow skin; headache while the fever lasted; very weak after the paroxysm. I gave Chin-sul.$^{1x}$, one powder every hour; called the next day; no change. Take Chin-sul.$^{1x}$. Continued Chin-sul., first, second, and third triturations, for a week. At this time she had a gone, sinking feeling during the chill which came at eleven A. M. as before. Fearing a severe congestive chill I gave Nux-vom.$^{3x}$. Called the next day at eleven A. M. and found her in a congestive chill from which she came very near dying.

This resulted in my discharge from a good homœopathic family and an allopathic physician called, who gave twenty grains of Quinine at a dose until he had suppressed the chills, making her sick all summer from its effect. My first experience with chills was a sad one. Perchance, a few days later, I was talking with Mr. B., who related his sufferings with chills a few years ago, incapacitating him from attending to his business all summer. Finally he called on a homœopathic physician, who gave him a high potency of some remedy which cured in a few days and he has never had a chill since. I said to myself this is the kind of Homœopathy that I am looking for; one such experience as I had just passed through was enough for me. I began to study *Allen on Intermittent Fever*, which is worth ten times its weight in gold to any physician who is living in a malarial district. By studying it four and a half years and putting its teaching to a practical test I have cured over five hundred cases of chills and reaped a rich harvest. I soon learned the importance of giving the indicated remedy in the minimum dose. If I had recorded all of my cases I could now report over five hundred cases cured with a high attenuation of remedies which proved to be the simillimum. Nat-mur. was indicated and cured oftener than any other remedy, of course, high; no man can ever persuade me to give low again for chills. What is true of chills is also true of any other disease. Last fall I had the pleasure of seeing three cases of typhoid malarial fever recover from the administration of Bry., Bapt., China, and Rhus-tox. high, while my allopathic brothers lost all they treated of the same disease. To prove the efficacy of the minimum dose to those who wish to try it and also to establish confidence in the minds of those who first prescribe the high with fear and trembling, as I did when I began practice here, I will report a few cases recorded and from letters I hold of cases treated by mail.

CASE 1.—Miss R., aged seven years, stout and healthy in appearance; had an attack of chills. Mother writes thus: "My girl had a chill at half-past seven A. M. to-day, complained of being tired before chill, which lasted until nine A. M., duration of fever about three hours, which was so severe she was out of her head most of the time. Chill beginning in the back, coldness of the hands and feet, no appetite, bowels constipated, sweat scanty, severe throbbing headache, better when begin to perspire."

I sent Nat-mur$^{200}$, a powder every two hours. I got a letter the third day stating the chill came at half-past nine A. M., but not so hard. Sent Sac-lac.

The sixth day I received another letter saying Mona had a very light chill; continued Sac-lac. one week, then take one powder Nat-mur.$^{200}$; has had no more chills now for three years. One week later Mr. R. sent his hired man to me for treatment.

CASE 2.—A young man, aged about twenty-two years, had chills four months every other day. "Taking Quinine and having the chills was his best hold." He had very few symptoms other than a proving from Quinine. I prescribed on three symptoms, stomach bloated with belching, bowels constipated, sweat on being covered, and the sweat parboils the skin. I gave him six powders of China$^{200}$, and told him if he would take one powder three times a day until gone he never would have another chill. Have seen him once and Mr. R. twice since, and both said he never had another chill for three years.

CASE 3.—Rev. W., aged thirty-five years, was taken with a chill at San José, on his way to Conference at Monterey. A homœopathic physician was called in and gave Chin-sul.$^{1x}$, later Nux-vom.$^{2x}$; no change except the doctor got a proving of Nux, and had it not been for his patient's kind forbearance, probably saved an uprising which might have resulted in a cool reception. In three days I received a telegram which read thus: "Mr. W. had a severe chill at half-past twelve M., terrible burning pain in his stomach, with great anguish, high fever, very restless and frequent drinking." I telegraphed take "Ars.$^{200}$ every hour for twelve hours." He had no more chills for two weeks, and then the chills returned, caused by taking a midnight repast. Ars. cured promptly, having only two light chills since, now over three years.

CASE 4.—Mr. B., aged thirty years, strong constitution, has been shaking five months, a chill every other day at three P. M., and a good picture of Quinine. Gave Nat-mur.$^{200}$, six powders, one every three hours. The next chill was the hardest he ever had. At ten A. M. two more light chills ended the series and Mr. B. has had no more chills for four years. On investigation I learned that Mr. B. had had his first few chills at ten A. M., and only when he began to take Quinine the time changed. He had such a strong desire for salt he carried it in his pocket, and described his headache during the fever as though there was something thumping against his brain. I prescribed on the original symptoms, and developed the chills very similar to them as they began.

CASE 5.—Mr. H., aged forty years, a full-dosed allopathic patient, sent for me to cure the chills; has been taking Quinine in large doses for four and a half months and chills, all of that time, every twenty-one days, making him so weak he was unable to work in the interval. I found him shaking, the chill began at half-past eleven A. M. His symptoms were another picture of Quinine. I told him if he was willing to stay in bed and have chills for a week I would cure him, and that I could not do it without making him have a few chills; he said anything to get cured. I traced his case back to the first chills and learned that his chills began at seven A. M., with very severe bone pains and bilious vomiting. Gave Eupat-per.$^{6x}$ every hour for two days, then Sac-lac. He had three severe chills and four light chills the next seven days, the eighth chill was only a coldness. He got up and went to work in a few days and has not had a chill since, now four years.

CASE 6.—An old lady consulted me about her grandchild, aged ten years. She was nearly deaf, only could hear when spoken to in a high tone of voice. She had good hearing before she took Quinine for chills.

Before she took Quinine her chills came in the morning—I could not learn the hour. Gave Eupat-per.$^{6}$ every hour for two days. The second day after taking the medicine, at 8.30 A. M., she had a severe chill. She had bone pains, pains in her stomach, vomited a bilious matter of a yellowish-green color. She was curled up in bed, very thirsty; high fever followed the chill, continuing about three hours; muttering delirium, very red cheeks, very restless and irritable, severe headache, which lasted all day, sweat scanty. Three more doses of Eupat-per.$^{6}$ and Sac-lac. for one week. She had three more chills, each chill coming in the morning about the same hour as the first. She has never had a chill since, and in three weeks after she had the chills she could hear as well as ever.

CASE 7.—Mr. O. writes thus: "My wife (aged twenty-five) had a chill Friday, June 14th, 1889. Severe pains all through her bones, especially ankles, which give out, will not hold her up. Pains in legs, back, side, back of head, and frontal headache, burning pains in back of the eyes, eyelids hot. She cannot keep the eyes closed or open; creeping chills up the back, with some coldness of the extremities. To-day's chill, June 16th, came at half-past seven A. M.; numbness of the hands and feet, chill continued one hour, then fever of three hours' duration. Severe pains in bones during chill, pulse one hundred and twenty, strong and regular. Pulse at half-past eleven A. M. one hundred and forty. Splitting headache, burning pains back of head and through the eyes, headache so severe she cannot keep quiet in any position; cries a good deal. Faintness, nausea, and vomiting; both sides of her neck very sore and stiff, hurts to move her head. Soreness, pain, and lameness in left side, just under ribs, extending to the right side. Severe pains in the cords of the neck; mouth tastes gluey and sweet, fever sores coming out all around her mouth; urine frequent and of an orange color, burning hot and painful; pain through small of back and kidneys. Face pale and sallow, dark circles around her eyes; not much thirst, drinks once an hour, water has no taste."

I sent Nat-mur.$^{200}$, one powder every hour. She got the medicine Monday, June 17th, at half-past eleven A. M.

She had a light paroxysm the next morning and none since, now eight months.

"Folsom, June 22d, Dear Dr.—My wife has not had a chill since Tuesday. She did a big washing yesterday and ironing to-day. She has been dieting regularly on cucumbers and summer squash."

CASE 8.—Mr. M., aged about forty years, consulted me three years ago; said he was suffering with dumb ague every afternoon, at five P. M., he had a coldness, severe pain in his stomach; if he ate supper he was sure to vomit, therefore he abstained from eating his last meal each day. He had a bilious diarrhœa and some fever; said he has been taking Quinine all summer. I gave Ars.$^{3x}$, China$^{3x}$, Chin-sul.$^{200}$, Puls.$^{3x}$. Ars. relieved the pain in the stomach to a great extent, and there was a diminution of the passages from the bowels. He thought, after trying Homœopathy two weeks with only an amelioration of two symptoms, he would go back to allopathy, which he did in spite of my promises to relieve him in a few days. Last September his wife came into my office and said her husband had been suffering with chills every year since I treated him three years ago; she also stated that they had got disgusted with this locality and they were going East just as soon as her husband is well enough to go. She inquired of me if I could do anything for him, "if so come up soon as you can." In one-half hour I was at his bedside. One glance at his face proved to my mind that he had been dieting regularly on the three staple meals of allopathy—viz.: Quinine, Fowler's solution, and Calomel. He admitted that he had continued this diet for three years faithfully, and what troubled him most, it did not benefit him any, although recommended by every physician in Marysville he had tried. I recognized the fact that I had a desperate case before me. He was pale, sallow, eyes sunken, face pinched, skin had a grayish tinge, weak, great prostration, flushes of heat and cold, hands and feet cold, chilly on the least exposure, tongue thickly furred with a yellowish coating and very dry, especially at night; severe headache, diarrhœa, twenty-five or thirty stools every twenty-four hours, stools watery and dark-brown color. Anus standing wide open, weak after stool; has had a stool every hour for three weeks.

Gave Phos.$^{30}$ in water every hour; next day anus not so relaxed and dilated; continued Phos.$^{200}$. Third day anus closed, and not quite so many stools. On the night of the third day, after taking Phos. at one A. M., he had a severe chill, high fever, frequent drinking, and very restless until morning; sweat scanty. Gave Ars.$^{200}$; two more chills at the same hour, but not so hard. The chill ceased, but the diarrhœa continued without any change; very weak; exhausted by the least movement; burning of anus after stool, an effect of Ars.; did not have burning of anus before taking Ars. I was about to give a higher attenuation of Ars., when he exclaimed: "Doctor, is it not peculiar? when I drink water, I must put some salt in it to make it taste right." I did not give Ars., and went to my office and got Nat-mur.$^{200}$, and gave it every hour in water. In two days the twenty-five or thirty stools a day had decreased to two a day. The fourth day he took a walk, and in a week he went to work. I never saw any one gain as fast as he did under the circumstances. Two weeks after he got to work he began to have bone pains, weak and nauseated. I told him he had not got all the malaria out of his system which had been suppressed by Quinine. Gave him Nat-mur.$^{200}$ for two days. The third day he had a chill at six P. M., and a chill every day for a week; second chill at five P. M.; the third and fourth chills at three P.M.; fifth, sixth, and seventh chills from twelve M. to one P.M. As the last chills were getting worse, I discontinued Sac-lac. and gave Ars.$^{200}$. The time of the chill and the restlessness, the nausea and burning pain in the stomach were sufficient reasons for interfering with my last prescription (Nat-mur.$^{200}$) and giving Ars.$^{200}$, which cured promptly, my patient having only one more light chill. He began to gain strength rapidly; now he is a picture of health, very fleshy. He asked me a few days ago if I had some anti-fat. He is an enthusiast on Homœopathy; says he is well satisfied to live in Marysville now.

CASE 9.—Mr. G., aged about thirty years, came direct from Vermont—a locality where malaria never was known. Two days after arriving here he was taken with a severe chill at ten A.M. Chill runs up and down his back; icy coldness of hands and feet: headache soon after chill begins, and continues until he has perspired two hours. Sweat profuse. He had what he described as a hammering headache; high fever continuing six hours. The profuse sweat relieved him of all pains; very weak after paroxysm. Gave Nat-mur.$^{200}$ every two hours in the absence of the paroxysm for two days. One more chill ended his shaking. About two months ago I received a letter from a friend, a homœopathic physician in the East. He said he used the homœopathic remedies very low, and he is very well satisfied with the results. There are indolent Oriental people in this enlightened and civilized age in which we live who use a crooked stick for a plow, and they are satisfied with their mode of farming. Mr. G. received enough of the poison of malaria sleeping on the second floor, above the point where it is said the malarial poison rises. How wonderful—nevertheless true—he received by olfaction enough of the malarial poison to produce two severe chills! My friend would probably say, how much more wonderful that the two hundredth potency of Nat-mur.$^{200}$ cured in two days.

The dynamics of Nat-mur.$^{200}$ must have been stronger than the malarial poison, or it would not have cured. I prescribed the two hundredth attenuation in the majority of my cases of chills. I have cured, as I have said, five hundred cases, but a few remedies higher. I am convinced the highest attenuations would have done better. Every homœopathic physician knows how important it is to thoroughly individualize his cases in order to get the right remedy. Every homœopathic physician should most emphatically know the importance of giving the minimum dose. The mode of giving the indicated remedy in the highest attenuations, and never to repeat as long as there is improvement has cured more chronic cases hitherto considered incurable, and has turned out more enthusiasts on Homœopathy than any other mode. My dear brother, you who are satisfied with the crooked stick for a plow, to continue to live in a crude atmosphere, and to be influenced by your environments of crude dosing, may I hope that some time, amid your discouragements, you may throw aside prejudice and try the high and highest attenuations. After giving them a fair trial, you will be convinced that the high is the longest and deepest acting, and also cures more completely and permanently.

